# A randomised controlled trial to investigate effects of enhanced supervision on primary eye care services at health centres in Kenya, Malawi and Tanzania

**DOI:** 10.1186/1472-6963-14-S1-S6

**Published:** 2014-05-12

**Authors:** Khumbo Kalua, Michael Gichangi, Ernest Barassa, Edson Eliah, Susan Lewallen, Paul Courtright

**Affiliations:** 1 Department of Ophthalmology, University of Malawi, College of Medicine, Blantyre, Malawi; 2 Blantyre Institute for Community Ophthalmology, Lions Sight First Eye Hospital, Blantyre, Malawi; 3 Ministry of Health, Kenya; 4 Kilimanjaro Centre for Community Ophthalmology Tanzania, PO Box 2265, Moshi, Tanzania; 5 Kilimanjaro Centre for Community Ophthalmology International, University of Cape Town, South Africa

**Keywords:** primary eye care, supervision, Kenya, Malawi, Tanzania, human resources, soins de la vue primaires, supervision, Kenya, Malawi, Tanzanie, ressources humaines

## Abstract

**Background:**

Knowledge and skills of primary health care workers (PHCWs) in primary eye care have been demonstrated to be inadequate in several districts of Kenya, Malawi, and Tanzania. We tested whether enhanced supervision, focused on improving practical skills over two years, would raise the scores of these workers on a test of basic knowledge and skills.

**Methods:**

This was a randomised controlled trial. All primary health care (PHC) facilities within two districts of each country were enrolled and randomly assigned by district (Kenya, Malawi) or by health care facility (Tanzania) to receive quarterly skills-based supervision by a district eye coordinator or to continue existing routine supervision. At baseline, a test of basic knowledge and skills in five key areas was administered to PHCWs, and visual acuity (VA) charts and working torches were provided. After two years the test was administered again. Changes in test scores were compared between the intervention (enhanced supervision) and the non-intervention (routine supervision) facilities.

**Results:**

All 137 facilities in the six districts were enrolled including 343 PHCWs. At baseline, no facility had a visual acuity chart and 18 (13%) had a working torch; the average total skills scores were 6.04 and 6.38 (maximum of 12) in the non-intervention and the intervention facilities, respectively. After two years, 16 intervention facilities (23.2%) had a visual acuity chart correctly placed and 19 (27.5%) had a working torch, compared to 4 (5.9%) and 6 (8.8%), respectively, in the routine supervision facilities. At the facility level, the change in overall test scores was +1.84 points in the intervention sites compared to +0.42 points in the non-intervention sites (p<0.001). Staff turnover included about 75% of the staff by the end of the study.

**Conclusion:**

The improvements in the enhanced supervision facilities were very modest and of questionable clinical significance. The low impact of the intervention may be due to the high turnover of PHCWs or high absenteeism. A better understanding of the quality of eye care at PHC facilities and influencing factors are urgently needed before continuing to invest resources in the scale up of this model of task shifting in Africa.

## Abstract (fr)

**Résumé:**

**Contexte**

Dans plusieurs districts du Kenya, du Malawi et de la Tanzanie, les connaissances et compétences des fournisseurs de soins de santé primaires en soins de la vue primaires sont inadéquates. Nous avons procédé à une évaluation pour savoir si une amélioration de la supervision visant à renforcer les compétences pratiques sur une période de deux ans permettrait à ces travailleurs de mieux réussir un examen sur leurs connaissances et compétences de base.

**Méthodes**

Nous avons réalisé un essai comparatif avec répartition aléatoire. Tous les établissements de soins de santé de deux districts de chaque pays ont été répertoriés et choisis de façon aléatoire en fonction de leur district (au Kenya et au Malawi) ou sans égard à leur district (en Tanzanie) pour recevoir – ou non – une supervision trimestrielle axée sur les compétences assurée par un coordonnateur de district en santé de la vue. Au préalable, un examen des connaissances et des compétences pour cinq tâches clés a été distribué aux travailleurs. On leur a aussi fourni des grilles d’évaluation de l’acuité visuelle et des lampes de travail. Au terme d’une période de deux ans, les travailleurs ont été invités à refaire l’examen. Les établissements sujets (où l’on a amélioré la supervision) et les établissements témoins (où la supervision est demeurée la même) ont été comparés en fonction des différences observées entre les résultats de leurs travailleurs respectifs aux deux examens.

Au total, 137 établissements comptant 343 fournisseurs de soins de santé primaires ont été répertoriés dans les six districts compris dans l’essai. Initialement, aucun établissement ne possédait de grille d’évaluation de l’acuité visuelle, et 18 (13 %) d’entre eux seulement possédaient une lampe de travail. Les résultats moyens à l’examen étaient de 6,04 et de 6,38 (sur 12) dans les établissements témoins et sujets, respectivement. Après deux ans, 16 établissements (23,2 %) sujets possédaient une grille d’évaluation de l’acuité visuelle placée correctement et 19 (27,5 %), une lampe de travail, contre 4 (5,9 %) et 6 (8,8 %) établissements témoins. À l’échelle des groupes d’établissements, la différence entre les résultats aux deux examens était de +1,84 dans les établissements sujets contre +0,42 dans les établissements témoins (p<0,001). À la fin de l’étude, environ 75 % des travailleurs n’étaient plus à l’emploi des établissements.

Les améliorations observées dans les établissements sujets sont très modestes, et leur signification sur le plan clinique est discutable. Les maigres résultats de l’intervention peuvent être attribués au roulement élevé ou au haut taux d’absentéisme du personnel des soins de santé primaires. Avant de continuer à investir des ressources pour faire passer à grande échelle ce modèle de délégation de tâches en Afrique, il faut d’abord acquérir une meilleure compréhension de la qualité des soins de la vue offerts dans les établissements de soins de santé primaires et des facteurs qui influent sur elle.

## Background

Primary eye care (PEC) in sub-Saharan Africa usually refers to the provision of basic eye services (diagnosis, treatment, and referral) integrated within the primary health care (PHC) system, offered at the most basic level of the health system by general primary health care workers (PHCWs). It is estimated that only 30% of Africans have access to specialised eye care at any point and therefore, by default, most patients are attended by frontline health workers, who may not have received any training in eye care [1]. An important argument in favour of using PHCWs to provide basic eye services comes from evidence that integrated health systems, rather than fragmented health service, are a more cost effective method [2] of delivering health services..

Therefore, it is often assumed that eye care services can only be made sustainable if they are integrated into the basic health services. The assumption is also made that if eye care is provided at the basic health care level it will increase accessibility for the rural poor. On the other hand, there are arguments that PEC will not be an effective way to reach the goal of elimination of blindness and visual impairment because cataract, glaucoma, and refractive errors are the most common causes for blindness and visual impairment in Africa and these conditions, unfortunately, cannot be treated at the level of primary care system in Africa; in fact it is a challenge to even diagnose them accurately without more technical knowledge and equipment than are present at the primary health care level. It should be also be considered that there might be lower quality when specialized services are delivered by general health workers. In the case of eye care, there could be many reasons for the low quality, including inadequate training and supervision, workload, and infrequent encounters with patient with eye complaints, among others. Supervision in particular has been noted to be a problem at PHC facilities in Tanzania [3]. Ehiri suggested that poor quality in primary child health services in Nigeria could be attributed to inadequate facilities and personnel as well as failure to use clinical protocols and lack of systematic supervision [4]. Rowe et al, proposed that health worker performance is rarely improved by dissemination of written guidelines, but that supervision and audit were often effective [5]. A pilot study in one district of Tanzania documented the inadequate skills of PHCWs in PEC [6].

The challenges of integrating health services have included severe shortage of human resource capacity to implement the programmes and scale up [7], lack of personnel with skills in specialized areas, difficulty to supervise and monitor activities, large financial costs for professional development of disease-specific programs due to continuous change of staff roles, and staff being resistant to integration [8]. Health workers are both in short supply and poorly distributed in developing countries. A potential solution that has been suggested is so-called “task shifting”; this means shifting tasks from workers who are highly specialized (and presumably scarce and costly) to those who are less specialized (and presumably more abundant and less costly to employ). In eye care, task shifting of surgical procedures has been used for two specific conditions. One is for cataract surgery where some countries have trained a cadre of non-physician cataract surgeons to deal with uncomplicated cases. The second example is shifting the task of trichiasis surgery from ophthalmic clinical officers and nurses to general health workers. Primary eye care can also be considered as a form of task shifting, wherein not one specific task but several activities are shifted to the primary care level, away from specialist cadres such as ophthalmic clinical officers and nurses. Although not formally defined, these primary eye care activities are supposed to cover whatever is necessary to manage anyone who presents with an ‘eye complaint.’ Management might include counselling, treatment, or referral to more specialised workers. In considering the concept of PEC today, it is useful to understand how it has evolved. The historic meeting in Alma Ata described the theory of primary health care (PHC). Those in the field of blindness prevention recognized two important causes of blindness in developing countries that could be reduced by efforts at the primary health care level: these were vitamin A deficiency-related blindness and trachoma. The former could be reduced through measles immunization and vitamin A supplementation; and the latter reduced through community based efforts at improved hygiene such as face washing and environmental improvements [9-11]. The concept of primary eye care expanded in the 1980s when ophthalmic workers recognized that an advanced (white) cataract was identifiable, even with no equipment, as was a red eye [12]. These signs on their own do not necessarily determine who needs referral and who does not, but combined with a measurement of visual acuity of the eye, they are helpful. Thus, the need to teach general health workers to measure visual acuity was added to the skills mix required for PEC. Tetracycline eye ointment was added to the standard list of medicines for primary health facilities since it is an effective treatment for active trachoma as well as some other infectious eye conditions. As activities aimed at reducing blindness and visual impairment increased globally, the expectation grew that workers on the front line could identify and treat eye problems in the community, or at least refer them appropriately to specialist eye workers.

There is a need to critically look at the available evidence of successes of existing primary eye care programs that have been integrated into PHC before further investments into PEC are made. A detailed review by Courtright *et al*[13] identified only five articles providing information on the effectiveness of general primary health care workers in practicing primary eye care. Steinkuller, in 1987, commented on the results of PEC training for general health care workers in South Africa, Tanzania, Kenya, and Malawi [14]. He acknowledged that they had some success in promoting general health messages, including the messages related to hygiene and nutrition information useful in reducing eye problems due to trachoma and vitamin A deficiency. He was not positive about their ability, in spite of PEC training, to diagnose and manage eye problems, specifically to measure visual acuity, or accurately differentiate the various causes of a red eye, or safely remove foreign bodies [14]. In a study from Tanzania, Al-Attas *et al* found unacceptably long delays in accessing specialist emergency eye care services among patients with eye trauma [15]. These were often associated with having made several visits to the primary health care centre, where inappropriate advice was provided, or staff were absent altogether. In 2000 in South Africa, De Wet *et al* identified an awkward and difficult referral system, lack of the right medicines at clinics, poor knowledge of eye conditions, and inadequate communication within the referral system, as problems limiting the ability of general primary health care staff to provide good primary eye care [16]. Late presentation of children with cataract to tertiary centres has been associated with the inadequate ability of health workers at primary and secondary levels to provide accurate information to the families about the condition and the urgency of the problem [17]. A program in Rwanda was described in two articles [1,18]. In the program, general PHC workers were trained in PEC along with village volunteers. This resulted in an initial increase in usage at the health centres by people with eye problems, but this was not sustained once specialist ophthalmic personnel started to visit the centres to supervise. The village volunteers, aware that ophthalmic clinical officers would be present on certain dates, preferred to refer cases at those times so that they could be examined by specialists. This reflects a lack of confidence in the PEC provided by general PHC workers. It was also clear that health workers who had not received any PEC training were treating eye patients [1,18] once eye medicines were made available. The conclusion was that the results “were generally not encouraging” and that the primary eye care in this setting was not meeting the needs or expectations of the target populations. It was recognized that data were limited and more research was required. A study in Tanzania on knowledge, skills, and productivity in primary eye care among health workers found that there was poor understanding of basic ocular conditions among primary health care workers [19]. It is apparent from these articles that the absence of a clear definition of primary eye care, what should be integrated, and who should be tasked poses challenges to the concept of PEC in practice. With the exception of one study done in Zanzibar [20], the literature shows that PHC workers face considerable challenges and do not appear to be providing a high quality eye care service, with weaknesses in training and supervision identified as some key factors contributing to the poor results.

The current study was designed to test whether in comparison to “routine supervision”, “enhanced” supervision over two years (i.e. quarterly, skills-based supervision) would improve the knowledge and skills of primary health care workers in primary eye care in Kenya, Tanzania, and Malawi.

## Methods

This was a prospective randomised cluster study. In each country, two non-adjacent districts were randomly assigned to the intervention or non-intervention arm. All government primary health care facilities within each district were included. The intervention plan and tools were developed through a tool development training workshop (intervention plan) and pilot testing. Specific relevant conditions at the facilities, and measurements of the knowledge and skills of the PHCWs there were made at baseline, using a standardize questionnaire (described below). After collection of baseline information, a torch, batteries, and a visual acuity chart were provided to both intervention and non-intervention facilities. District Eye Coordinators (DECs) were trained in “enhanced” supervisory methods, provided with supervisory monitoring forms, as well as a minimal incentive and transportation money. The training was two days and included short didactic sessions followed by interactive sessions to practice and demonstrate competence. Role play was used to gain skills in supervisory challenges. The DECs were coached and expected to demonstrate how they would teach each of five subjects included in the PEC test (visual acuity measurement, conjunctivitis, cataract, presbyopia, and trauma). They were to make supervisory visits to each health care facility every quarter; each visit included a focus on one of the five subjects. Non-intervention districts were informed of the study, asked to continue the supervision system that was already in place, and were not given any supervisory monitoring forms or extra funds to make visits.

The randomization in Kenya and Malawi was at the district level. In Tanzania, randomization was by facility because the DECs felt it was not “fair” to randomize by district; special attention was given to instructing the eye coordinators in the strict need to avoid “spillover” in the supervision provided.

Ethical clearance was obtained in each country: from Tumaini University in Tanzania, and the Ministries of Health in Kenya and Malawi. Consent was obtained from all health workers who participated in the study.

Training of the DECs in supervision, and of the interviewers, took place in each country separately, after a meeting of principal investigators from each country to determine methods. Study coordinators in each country contacted the DECs by phone every quarter to ensure that supervisory visits were actually made.

Two questionnaires were used, both administered orally by the trained interviewers (research assistants). One pertained to conditions at the facility and included questions on staffing at the facility, as well as the availability of a working torch, tetracycline eye ointment, and a visual acuity chart. This was administered to the facility in-charge and took about 15 minutes to complete. The second questionnaire was administered to individual PHCWs. It was designed to test basic knowledge and skills; 8 points were allotted to testing ability to recognize (1 point) and manage (1 point) four common or important eye conditions (advanced cataract, conjunctivitis, presbyopia, and severe trauma) presented as cases with large colour photos and a brief history for each. Questions were mixed, some with yes/no answers and others needing numerical answers (year when trained, refreshed) and others asking specifically what topics were taught/learnt. The last part of the questionnaire was designed to measure specific clinical skills. Photographs and histories consistent with four important eye conditions (advanced cataract, conjunctivitis, presbyopia, and trauma) were shown; one point was given for recognition of the condition and another point for suggesting the proper management. Four additional points could be earned from demonstrating how to check visual acuity using a Snellen chart; one point was given for each of the following: correct distance, measuring each eye separately, recording the visual acuity, and interpreting what it meant. The test was simple and designed to explore a lower threshold for competence in recognition and management, based on the experiences of the authors. A total overall score was calculated by adding the above scores, with a maximum of 12 points possible. The pre-coded individual questionnaire took approximately 20 minutes to complete.

The questionnaires, which were translated into Swahili for Tanzanians and administered in English in Kenya and Malawi, were pre-tested on PHCWs in Tanzania. The questionnaires were administered by research personnel in each country, all of whom were trained to ensure standardization in data collection.

Knowledge and skills were analysed at the facility level using an average of the scores of all the PHCWs present at the facility on the visit; this was necessitated by the high rate of turnover of PHCWs between baseline and follow up and also took into account the cluster effect of the supervision on individuals at one facility. All staff present on the day of the visit were sampled.

Data were entered by double data entry in Epidata 3.1 (electronic software for data entry), which has inbuilt quality checks, and imported into Microsoft Excel and Stata 11.0 for analysis. We compared facilities pre-intervention using chi square or t-test. We calculated average scores for all PHCWs at each facility for recognizing and managing each condition, measuring VA, and total overall score. We analysed the change in total average score from baseline to follow up for each health centre and compared these changes by t-test between the intervention and non–intervention facilities. Changes in individual test items were analysed by a non-parametric (Kruskal-Wallis) test.

## Results

A total of 343 PHCWs from 137 health centres, 69 with enhanced supervision (intervention) and 68 with routine supervision (non-intervention), were studied. All supervisory visits to the intervention facilities occurred as scheduled except for two visits in Malawi which were not made due to unavailability of fuel. In Malawi there were 38 health facilities in total: 18 non-intervention, 20 Intervention; in Tanzania 58 in total: 29 non-intervention, 29 intervention; and in Kenya: 40 in total: 20 non-intervention, and 20 intervention.

There was a high turnover of PHCWs, with only 20% of the workers interviewed at baseline still in place at the end of two years; the positions of most of those who left had been filled by others. There was also high absenteeism with approximately 1/3 of all PHCWs absent during the two visits. One supervisor in Tanzania left his post halfway through the study and was not replaced by the Ministry of Health. Data from the facilities to which he was providing enhanced supervision were analysed with other enhanced facilities. Results were very similar in all countries so we elected to present them together.

A comparison of the facilities at baseline (Table 1) shows that they were very similar in terms of basic infrastructure, both in general and for eye care. None of the facilities had a VA chart. Working torches were present in 3 (5%), 4 (10%), and 4 (10.5%) of the facilities in Tanzania, Kenya, and Malawi, respectively. Tetracycline eye ointment was available in 50 (84.7%), 40 (100%), and 17 (44.7%) of the facilities in Tanzania, Kenya, and Malawi, respectively.

**Table 1:** Comparison of intervention and non-intervention facilities at baseline

Attribute	Number of intervention facilities with attribute	Number of non-intervention facilities with attribute	p-value
Improved road*	15/68	9/68	0.17
**Piped water or well**	43/69	43/68	0.95
**>1 hour from District Hospital in dry season**	27/69	34/68	0.20
**Main power**	44/69	43/68	0.95
Had ≥1 visit from DEC in previous 12 months*	31/68	29/68	0.73
**Number with functioning torch (%)**	2/69	11/68	0.02
**Number with VA chart correctly in place (%)**	0	0	NA
**Number with tetracycline eye ointment (%)**	51/69	56/68	0.23
**Mean baseline skills score on PEC test out of 12 possible points**	6.02 (SD 2.22)	6.38 (SD 2.01)	0.34

* Data missing from one non-intervention facility

Table 2 shows the status at follow up of the torches and VA charts that were provided to all facilities. The intervention groups were more likely to have a functioning torch (OR=3.9, 95% CI: 1.5, 10.6) and a visual acuity chart correctly placed on the wall (OR=4.8, 95% CI: 1.5, 15.3) than non-intervention facilities, although the majority of facilities no longer had these items. Tetracycline eye ointment was available at virtually all facilities, regardless of supervisory status.

**Table 2:** Number of facilities with basic PEC supplies at follow up

	With functioning torch (%)	No functioning torch (%)	OR (95% CI)	VA chart correctly in place (%)	VA chart missing or not in place (%)	OR (95% CI)	Tetracycline (%)	No tetracycline	OR (95% CI) p-value
Intervention facility	19 (27.5)	50 (72.5)	3.9 (1.5, 10.6) p=0.004	16 (23.2)	53 (76.8)	4.8 (1.5, 15.3) p=0.004	40 (100)	0	1.08 (0.07, 17.92) p=0.95

Non-intervention facility	6 (8.8)	62 (91.2)		4 (5.9)	64 (93.1)		37 (97.4)	1 (2.6%)	

Total	25 (18.25)			20 (14.60)			77 (98.7) *		

* Data missing for Tanzania, so n=78.

Table 3 shows the changes in knowledge and skills scores at facilities in intervention and non-intervention facilities for each component of the testing, and overall. With all three countries combined, the change in total score in the intervention facilities (1.84 points) was significantly larger (p=0.001) than in the non-intervention facilities (0.42 points), and this was mostly due to their improved ability to measure VA. Knowledge regarding cataract, conjunctivitis, presbyopia, and trauma did not improve significantly in the intervention facilities compared to non-intervention.

**Table 3:** Change in scores pre/post intervention

		Tanzania (n=28 non-intervention, 29 intervention)		Kenya (n=20 non-intervention, 20 intervention)		Malawi (n=18 non-intervention, 20 intervention)		All countries	
	Group	Mean (SD)	P value	Mean (SD)	P value	Mean (SD)	P value	Mean (SD)	P value*

**Cataract**	Non-intervention	0.44 (0.88)	0.06	-1.56(0.93)	0.52	-1.1(0.92)	0.01	0.11 (0.96)	0.58
	Intervention	-0.13 (0.83)		0.02(0.65)		0.55(0.31)		0.16 (0.73)	
**Presbyopia**	Non-intervention	0.56(0.74)	0.39	0.17(1.13)	0.01	0.21(0.58)	0.48	0.34 (0.85)	0.21
	Intervention	0.33 (0.81)		01.13(0.76)		0.39(0.49)		0.58 (0.79)	
**Trauma**	Non-intervention	-.19 (0.7)	0.28	-.08(0.68)	0.23	-.5(0.8)	0.41	-0.24 (.73)	0.11
	Intervention	-.03 (0.53)		0.16(0.33)		-.33(0.66)		-0.62 (.55)	
**Conjunctivitis**	Non-intervention	0.32 (0.98)	0.36	-.14(0.5)	0.15	0.14(0.66)	0.065	0.13 (0.80)	0.243
	Intervention	0.11(0.85)		0.24(0.6)		0.52(0.48)		0.26 (0.71)	
**Visual acuity**	Non-intervention	-0.27 (1.3)	0.01	0.5(1.7)	0.04	0.19(1.2)	0.83	0.09 (1.44)	<0.001
	Intervention	0.8(1.69)		1.52(1.24)		0.41(0.6)		0.90 (1.37)	
**Total**	Non-intervention	0.85(2.5)	0.62	0.28(1.7)	<0.001	-0.7(2.5)	0.028	0.42 (2.6)	
	Intervention	1.2(2.8)		3.0(1.54)		1.53(1.62)		1.84 (2.3)	0.0011

## Discussion

This study documents the persistent deficiencies in the diagnosis and treatment of common eye conditions by PHCWs in three countries, despite enhanced supervision by DECs over a two year period. A strong point of this study is that it was conducted as a randomised trial to try to ensure that potentially confounding factors were equally distributed between the two arms of the study.

Our study results highlight some of the major problems with PEC, which is supposed to be provided by PHC facilities in the countries under study. At baseline, the facilities were poorly equipped to do the most rudimentary examination of an eye, although tetracycline eye ointment was commonly available and used despite this. Neither this, however, nor any other eye medicine, should be dispensed without an examination of the eye. In a companion paper we documented factors associated with scores at baseline on the test of knowledge and skills [21].

The high staff turnover and absenteeism are indicative of the problems within the PHC systems of the countries included in our study. Failure to replace staff (such as the eye coordinator who was a supervisor) who leave for long periods of time weakens the systems. Weak systems make the provision of quality care, especially specialized care, more challenging, and also make it imperative that good quality supervision take place and include skills transfer.

The fact that enhanced supervision is significantly associated with having a working torch, and having a VA chart correctly placed on the wall, suggests that supervision helps. Even so, despite having been provided with these, only about one-quarter of the enhanced-supervision facilities had working torches and correctly placed VA charts after two years.

With regular supervision based on skills transfer, we demonstrated a modest increase in the ability of PHCWs to measure VA. This is a fundamental part of the examination of an eye but must be used in conjunction with history and further examination to determine appropriate management. While statistically significant, the improvements were of questionable clinical relevance and unlikely to be enough to actually lead to improved eye care in the health facilities.

There are a number of limitations in this study. While we believe that these facilities and the PHCWs within them are representative of others in the three countries, this cannot be proven. The personnel who collected data were trained to do so objectively, but it was not possible to keep them from knowing which facilities received intervention and which did not; this could have resulted in some bias. In Tanzania, the loss of a supervisor halfway through the study meant that half of the intervention facilities missed the enhanced supervision they should have received; if the supervision had been effective, the scores at these facilities might have been higher post intervention. However, this weakness in the health system is a reality that must be considered when task shifting strategies aimed at providing more services at PHC level are proposed.

An important consideration is the design of a good test of PEC knowledge and skills. There is no common understanding of what PEC means and thus the skills and knowledge required to provide PEC may be debated [22]. However, visual acuity measurement, basic eye examination, diagnosis, and referral have been suggested to be components of PEC [23]. Our test was based on these components, covering conditions that were either very common (conjunctivitis and presbyopia), the leading cause of vision loss (cataract) or requiring urgent referral (severe trauma). There is an urgent need to define more precisely what is meant by primary eye care, what skills are required to provide it, and how realistic it is to train general PHCWs to gain proficiency in the corresponding skills. A companion paper provides some evidence on the usefulness of ocular signs and symptoms commonly included in PEC training [24].

## Conclusion

In summary, we demonstrated a modest improvement in knowledge and skills in PEC after two years of skills-based supervision. It might have been more effective had the staff turnover not been so high. It is possible that if such supervision were provided uniformly throughout the health system, PHCWs would benefit even when transferred elsewhere. Good supervision need not be costly and should be part of routine activities. The fact that such supervision is not routinely practiced is evidence of the poor functioning of the current PHC system and leads to the bigger question of what is realistic to expect from PHCWs in terms of delivering specialist care of any type. For now, it is not clear that PEC, focused on diagnosis and management of eye conditions, will result in quality care for patients or that will it result in decreased prevalence of vision loss in the population, even with the implementation of enhanced supervision and oversight. This is not to say that there is no role for the PHCW in providing PEC; however, it might be better limited to knowledge of where to refer people they see with eye complaints, particularly in settings where few eye patients present to PHC facilities. More evidence on the effectiveness of current concepts of PEC is required before advocating for their expansion.

## List of abbreviations used

CI: confidence interval; DECs: District Eye Coordinators; DFATD: Foreign Affairs, Trade and Development Canada; GHRI: Global Health Research Initiative; IDRC: International Development Research Centre; KIs: village key informants; OR: odds ratio; PEC: primary eye care; PHC: primary health care; PHCWs: primary health care workers.

## Competing interests

The authors declare that they have no competing interests.

## Authors’ contribution

KK designed and conducted the experiment, analysed data, and contributed to write up. MG, EB, and EE conducted the experiment, and reviewed the manuscript. SL designed the experiment, analysed data, and contributed to write up. PC designed the experiment, analysed data, and reviewed the manuscript.

## Declarations

The publication costs associated with this article are funded by Foreign Affairs, Trade and Development Canada and the International Development Research Centre through the Global Health Research Initiative.

This article has been published as part of *BMC Health Services Research* Volume 14 Supplement 1, 2014: Uptake and impact of research for evidence-based practice: lessons from the Africa Health Systems Initiative's research component. The full contents of the supplement are available online at http://www.biomedcentral.com/bmchealthservres/supplements/14/S1
